# Supplementary figures and images for: A robust evaluation of TDP-43, poly GP, cellular pathology and behavior in an AAV-C9ORF72 (G4C2)66 mouse model
Source: Acta Neuropathol Commun. 2024 Dec 26;12:203. doi: 10.1186/s40478-024-01911-y (PMC11670477; doi:10.1186/s40478-024-01911-y)

**A**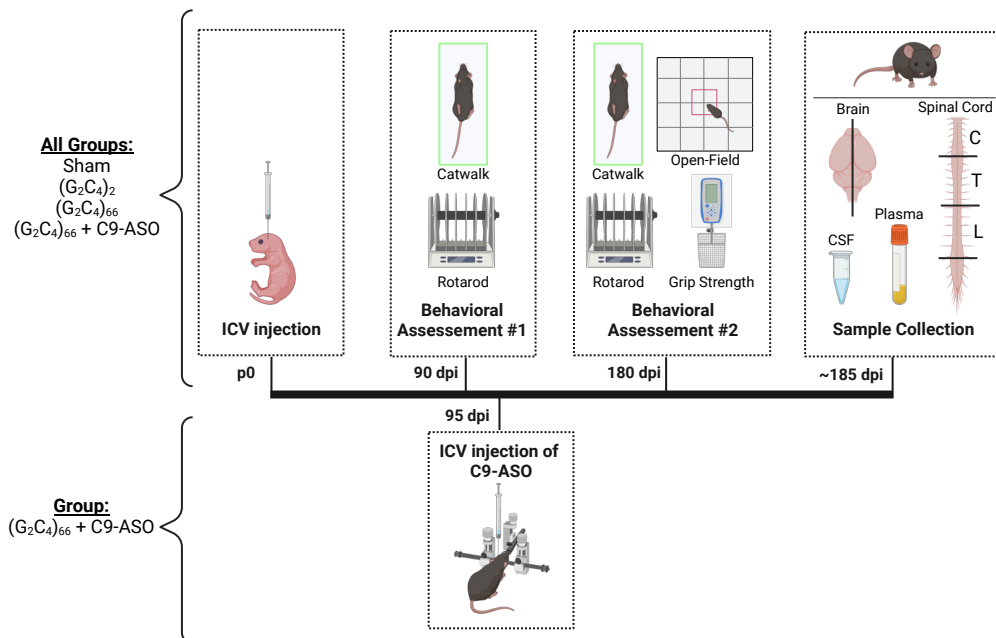**B**

### WPRE Expression Levels

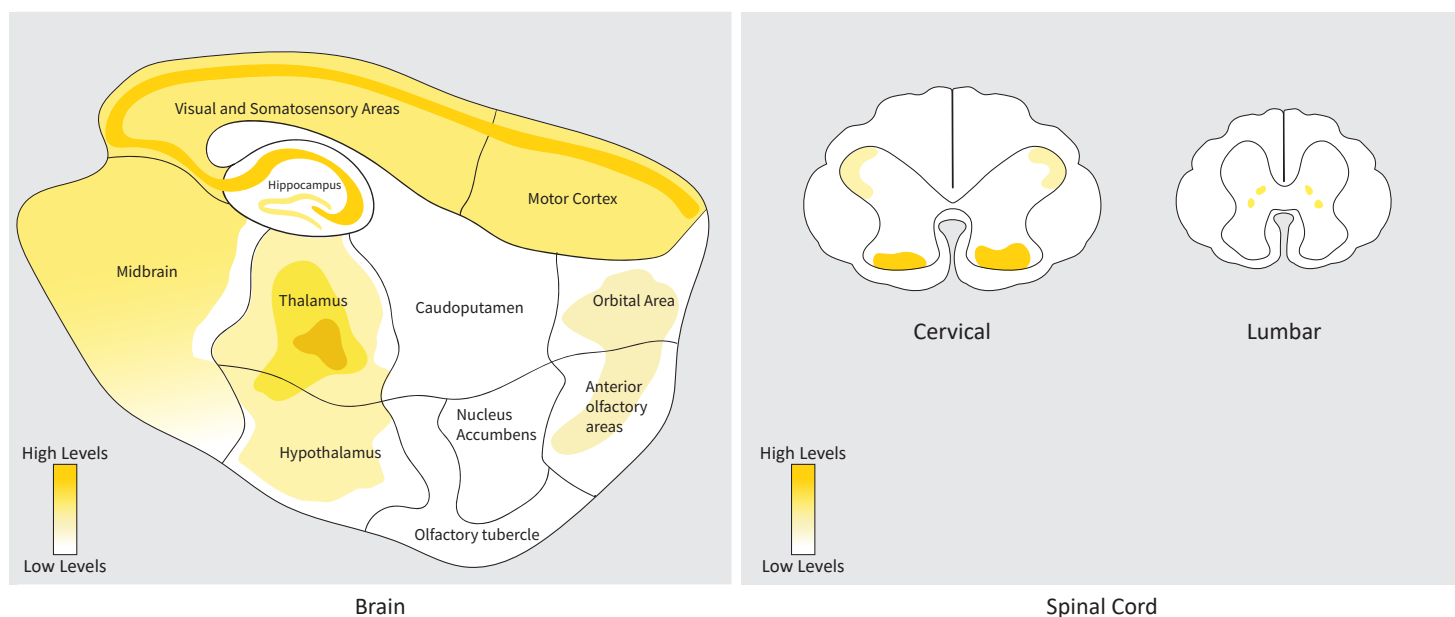**C**

### Protein Expression Levels

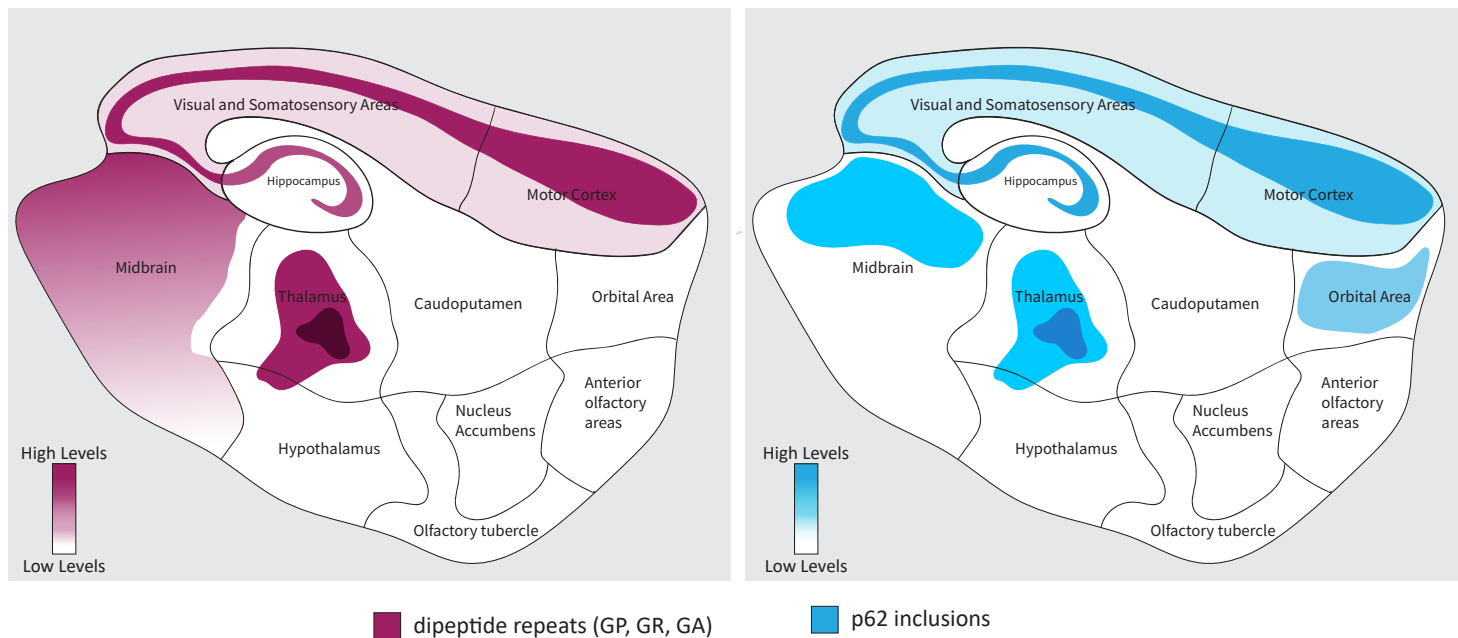

Supplement: Supplementary file 1 — Supplementary Material 1: Fig 1Schematic overview of the study design. Cartoon representation of spatial viral RNA expressionand protein expression levels of dipeptide repeat expression 33and p62 inclusionsin. Both DPR expression and p62 inclusions are localized to the areas where viral66 RNA is expressed [file 40478_2024_1911_MOESM1_ESM.pdf]

**A**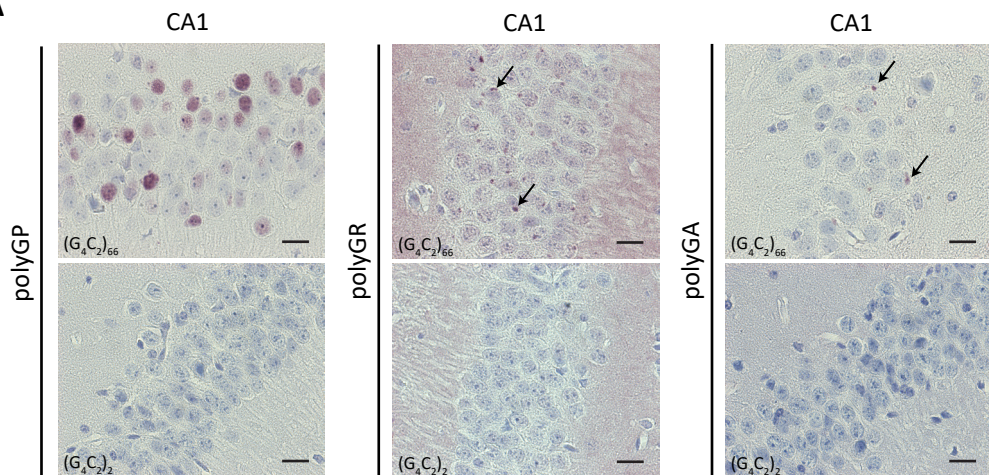**B**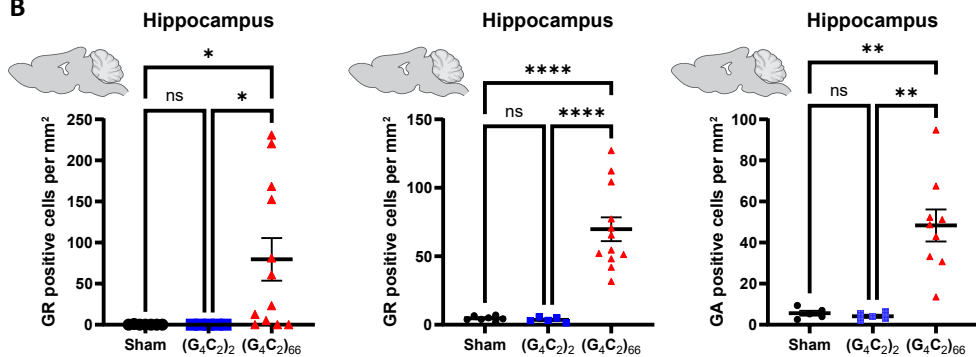**C**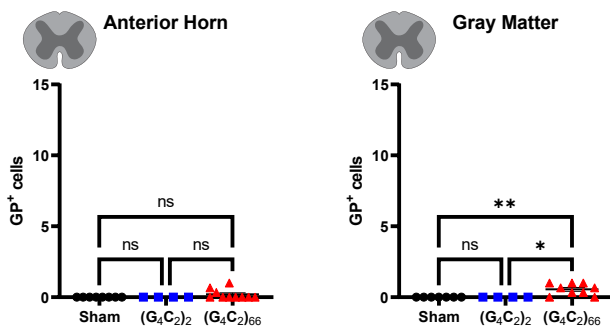

Supplement: Supplementary file 2 — Supplementary Material 2: Fig 266 mice have DPR expression in the hippocampus.Representative images of CA1 region from brain sections stained against polyGP, polyGR, and polyGA. Scale bars 20 μm.Quantification of sections stained against polyGP, polyGR, and polyGA2 n=12,66 n=12). One-way Welch ANOVA analysis of polyGP, polyGR, and polyGA positive cell number was performedfollowed by Tukey’s multiple comparison test. polyGP: sham vs2 p=0.9993, sham vs66 p=0.0303 and2 vs66 p=0.0304, polyGR: sham vs2 p=0.6542, sham vs66 p<0.0001 and2 vs66 p<0.0001, polyGA: sham vs2 p=0.6636, sham vs66 p=0.0018 and2 vs66 p=0.0013, error bars = SEM.Quantification of lumbar spinal cord sections stained against polyGP2 n=4,66 n=9 ). A Kruskal-Wallis test of the lumbar spinal cord gray matterand anterior horndataset was performed, followed by Dunn’s multiple comparisons test2 p>0.9999, sham vs.66 p=0.0064,2 vs.66 p=0.0301; anterior horn: sham vs.2 p>0.9999, sham vs.66 p=0.2180,2 vs.66 p=0.4504) , error bars = SEM [file 40478_2024_1911_MOESM2_ESM.pdf]

**A**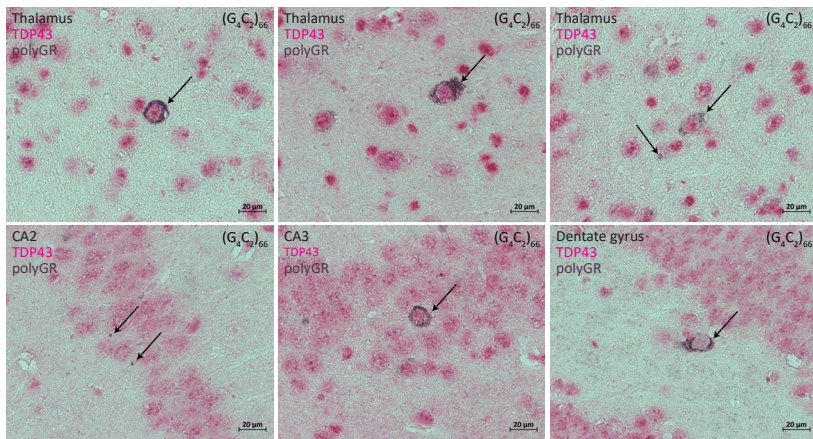**B**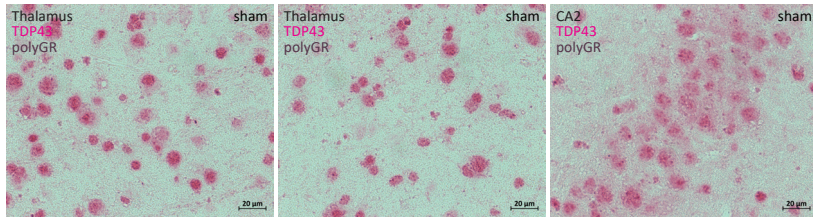

Supplement: Supplementary file 3 — Supplementary Material 3: Fig 3 TDP-43 does not mislocalize in polyGR positive cells.Representative images of Thalamus, CA1, CA3, and Dentate Gyrus brain regions in66 mice co-labled with TDP-43and polyGR. Scale bars 20 μm. No mislocalization of TDP-43 into the cytoplasm is observed in cells positive for TDP-43 and polyGR.Representative images of Thalamus and CA2 brain regions in sham mice co-labled with TDP-43and polyGR. No polyGR positive cells are present in sham tissue. Scale bars 20 μm [file 40478_2024_1911_MOESM3_ESM.pdf]

A

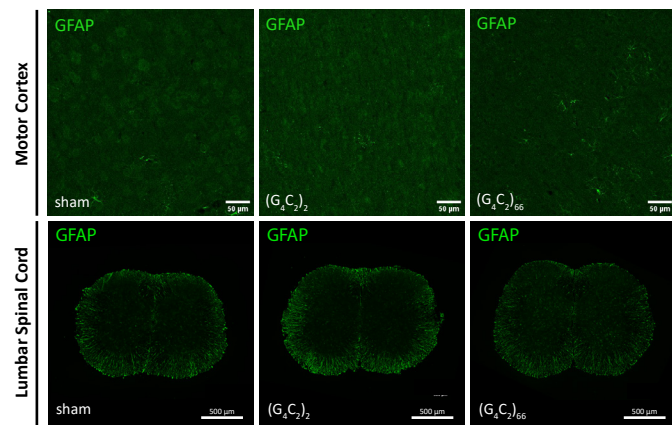

B

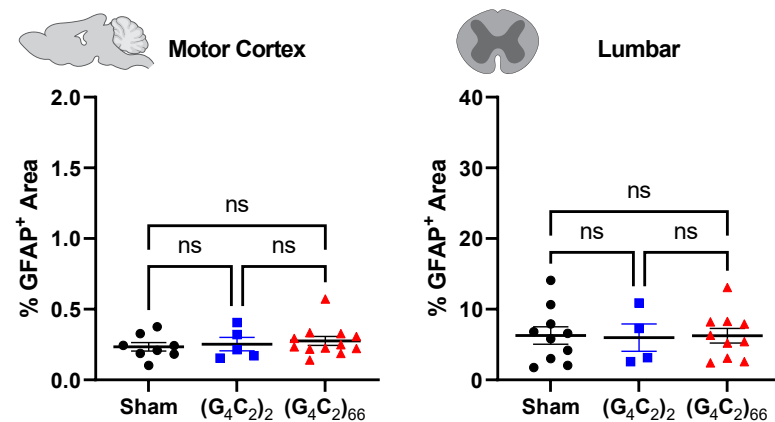

C

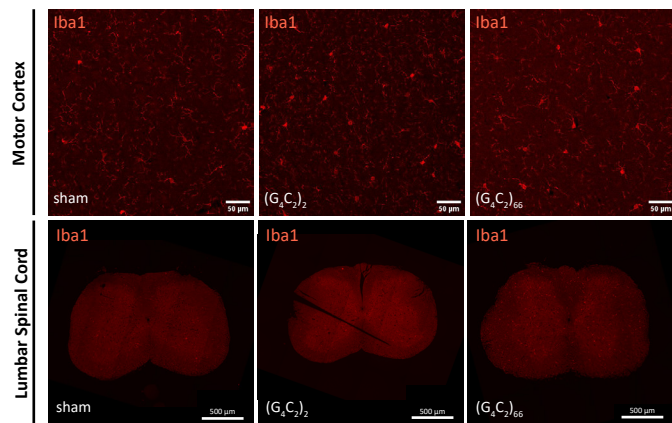

D

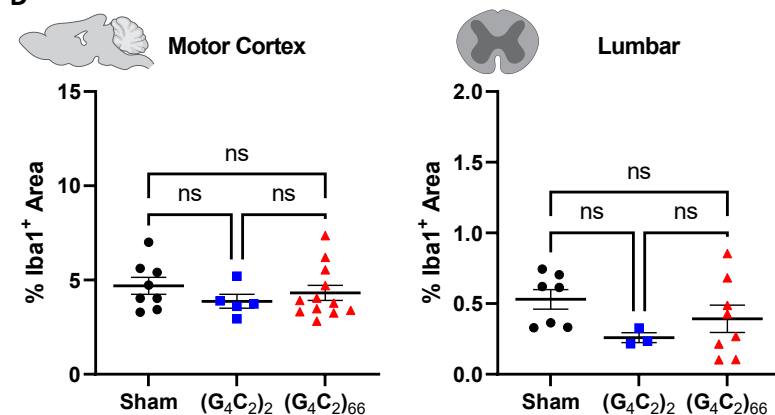

Supplement: Supplementary file 4 — Supplementary Material 4: Fig 4 Analysis of gliosis in the66 mouse model by immunohistochemistry.Representative images of immunohistochemistry analysis of GFAP expression within the motor cortex and lumbar spinal cord. Motor cortex scale bars 50 μm and lumbar spinal cord scale bars 200 μm.Quantification of % GFAP positive area within cortex and cervical spinal cord2 n=5 for, motor cortex and 4 for spinal cord,66 n=12 for motor cortex and 10 for spinal cord). One-way ANOVA analysis of the motor cortex dataset was performed, followed by Tukey’s multiple comparisons test: sham vs.2 p=0.9433, sham vs.66 p=0.6479,2 vs.66 p=0.9077. One-way ANOVA analysis of the lumbar spinal cord dataset was performed, followed by Tukey’s multiple comparisons test: sham vs.2 p=0.9888, sham vs.66 p=0.9998,2 vs.66 p=0.9909, error bars = SEM.Representative images of immunohistochemistry analysis of Iba1 expression within motor cortex and lumbar spinal cord. Cortex scale bars 50 μm and lumbar spinal cord scale bars 200 μm.Quantification of % Iba1 positive area within motor cortex and lumbar spinal cord2 n=5 for motor cortex and 3 for spinal cord,66 n=12 for motor cortex and 8 for spinal cord). One-way ANOVA analysis of the motor cortex dataset was performed, followed by Tukey’s multiple comparisons test: sham vs.2 p=0.4978, sham vs.66 p=0.7929,2 vs.66 p=0.7852. One-way ANOVA analysis of the lumbar spinal cord dataset was performed, followed by Tukey’s multiple comparisons test: sham vs.2 p=0.2085, sham vs.66 p=0.4674,2 vs.66 p=0.6513, error bars = SEM [file 40478_2024_1911_MOESM4_ESM.pdf]

A

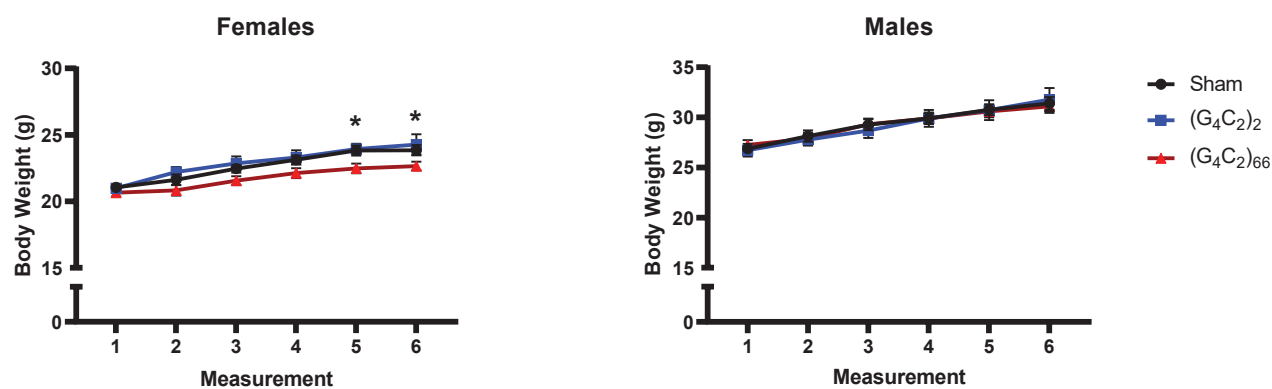

B

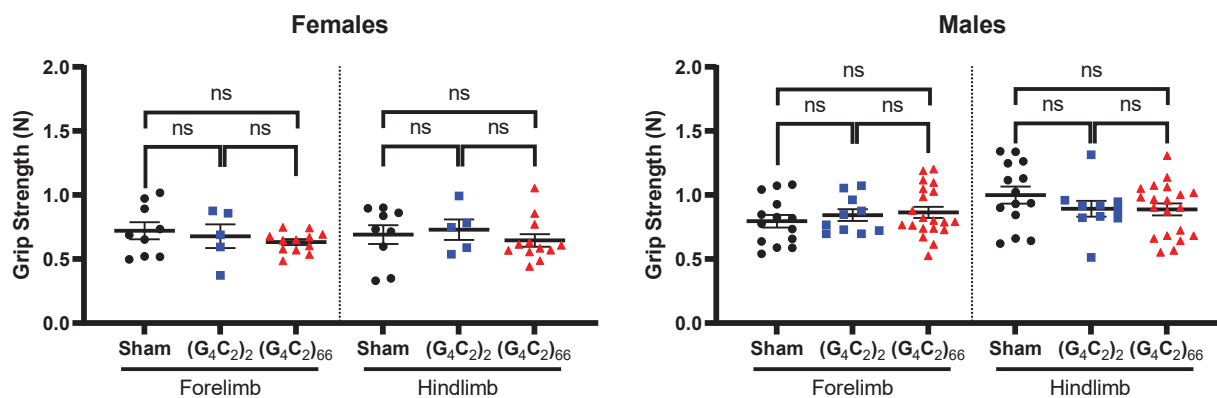

C

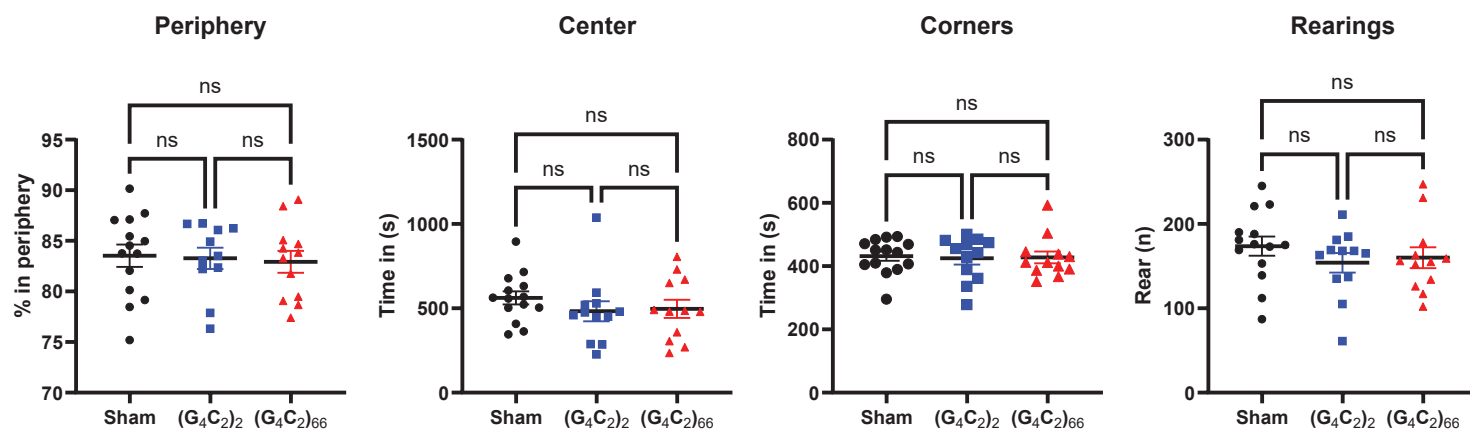

Supplement: Supplementary file 5 — Supplementary Material 5: Fig 566 mice do not have behavioral deficits.Bodyweight tracking of mice2 n=8,66 n=15, males: sham n=14,2 n=10,66 n=20). A two-way repeated-measures ANOVA analysis of the female dataset was performed, followed by Dunnett’s multiple comparisons test where a significant difference was observed at the fifth66 p=0.0276) and sixth sham vs.66 p=0.0479 measurement. A two-way repeated-measures ANOVA analysis of the male dataset was performed, followed by Dunnett’s multiple comparisons test but no significant difference was found between the groups for any of the bodyweight measurements, error bars = SEM.Grip strength behavioral analysis at 90 dpi in female2 n=8,66 n=12) and male mice2 n=7,66 n=20). One-way ANOVA analysis of the female dataset was performed, followed by Tukey’s multiple comparisons test: sham vs.2, sham vs. 3766,2 vs.66. One-way ANOVA analysis of the male dataset was performed, followed by Tukey’s multiple comparisons test: sham vs.2, sham vs.66,2 vs.66, error bars = SEM.Time spent in periphery, center, corners and number of rearing analyses in open-field test at 180 dpi2, n=1266). One-way ANOVA analyses of time spent in periphery, in center, in corners, and the number of rearing were performedfollowed by Tukey’s multiple comparison test. Periphery: sham vs2 p=0.9840, sham vs66 p=0.9130 and2 vs66 p=0.9741, Center: sham vs2 p=0.5099, sham vs66 p=0.6300 and2 vs66 p=0.9807, Corners: sham vs2 p=0.9620, sham vs66 p=0.9864 and2 vs66 p=0.9941, Rearings: sham vs2 p=0.4598, sham vs66 p=0.6833 and2 vs66 p=0.9339, error bars = SEM [file 40478_2024_1911_MOESM5_ESM.pdf]
